# Multiplication of the Unfit

**Published:** 1921-03-26

**Authors:** 


THE PUBLIC WELL-BEING—*(continued)*.

### Multiplication of the Unfit.

### AMERICA'S REMEDY.

We are getting on. It has just been announced that the Toronto Provincial Government intends to introduce in the present Legislature a Bill which would compel applicants for marriage licences to show that they are free from disease. That any Legislature should frame such a measure is not only evidence of considerable courage, but probably an indication that Canada's attitude on questions of eugenics is already rather more advanced than public opinion in the Old Country on this matter. It is, perhaps, as well to recognise frankly that Great Britain is not ready to adopt so drastic a measure as the introduction of compulsory certificates of health before marriage; one can imagine, indeed, the chorus of execration that would arise if the Minister of Health were to present a Bill on these lines to the House of Commons. The chorus would no doubt be equally devastating if the solution recently put forward by Mr. Harold Cox for preventing the multiplication of the unfit were offered from an official source. Mr. Cox based his argument on the text of a case in which a London magistrate a few weeks ago thus addressed the husband of a woman who had been brought before him on a charge of stealing: "Yours is a very sad case," said the magistrate. "It is an appalling thing for the future of this country and the race that people like you and your wife, who both seem to be suffering from every known disease, should grow up, marry, and have children."

He elaborates the familiar problem of the feeble-minded and their children, often carefully kept alive by the State, and points out how similar circumstances apply to persons suffering from "hereditary diseases," such as consumption or syphilis. At enormous expense the country maintains hospitals and other institutions for treating these diseases. Patients can generally obtain gratuitous, or almost gratuitous, treatment for an indefinite period. Yet if any such patients recover sufficiently to take their discharge they are free to have as many children as they choose, and to pass on to third and fourth generations the racial defects of the parents. The abstract proposition, therefore, is that if the community, at its expense, keeps alive and tries to cure an individual suffering from diseases of body and mind which are known to be heritable, it is justified in saying to those individuals that they should not pass on the taint of their blood to another generation.

### Mr. Harold Cox Quotes California.

Permanent detention of the unfit must in the majority of cases be dismissed as out of the question, and Mr. Cox goes to America for a solution—by sterilisation. Early in the present century several States began to pass laws providing for the sterilisation of the mentally defective. Down to 1914 no fewer than twelve States had then passed laws dealing with the matter, but some of the laws were so recent that at the time of the report of the Eugenics Record Office of New York no action had taken place under their authority. Even in those States where the law had been in operation for some time experience tended to show that in the majority of cases it was not necessary to invoke the compulsory powers conferred by statute. For example, Mr. Cox quotes the following report from Dr. Hatch, general superintendent of the California State Hospital:—

> Our plan of proceeding with the work follows an agreement with the Secretary of the State Board of Health and myself that relatives, when possible, should be consulted, the operation explained to them, and their written consent obtained before the work was performed. . . . In a few rare cases we have operated against the wish of the patients. . . . Many of those operated on have been discharged and are living at home in comfort. As a general rule all are benefited to some extent by the operation. In some of the vasectomy cases but little improvement in the mental condition is to be noted. We endeavour to keep track of those who are discharged, and receive reports from time to time. We have found no ill-effects. No interference

**THE PUBLIC WELL-BEING**—(*continued*).

has been noted in the marital relations. In our experience in this State we find very much less trouble in obtaining consent of relatives at the present time than when we first commenced the work. It is apparent that the public are being educated up to the value of the work.

This is an interesting report, and we hope Mr. Cox will be able to secure others of results up to and including the post-war period. But it seems to us useless at the present time to advocate an immediate adoption of such measures of prevention in this country. Even in their limited application the American methods suggest a certain danger to the liberty of the subject. That is an impression which will at once strike the average man in Great Britain. Education has a long way to go before the British public will swallow the logical remedy which Mr. Cox strongly advocates.

